# Progesterone Induces Apoptosis and Steroidogenesis in Porcine Placental Trophoblasts

**DOI:** 10.3390/ani12192704

**Published:** 2022-10-08

**Authors:** Yueshuai Liu, Hongxiang Ding, Yuze Yang, Yan Liu, Xin Cao, Tao Feng

**Affiliations:** 1 College of Life Science and Engineering, Northwest Minzu University, Lanzhou 730030, China; 2 Institute of Animal Husbandry and Veterinary Medicine (IAHVM), Beijing Academy of Agriculture and Forestry Sciences (BAAFS), Beijing 100097, China; 3 Joint Laboratory of Animal Science between IAHVM of BAAFS and Division of Agricultural Science and Natural Resource of Oklahoma State University, Beijing 100097, China; 4 Beijing General Station of Animal Husbandry, Beijing 100107, China

**Keywords:** progesterone, porcine placental trophoblast cell, steroidogenesis, gene expression

## Abstract

**Simple Summary:**

This study investigated the effects of progesterone treatment in vitro on apoptosis and steroidogenesis in porcine placental trophoblasts and the underlying molecular mechanisms. Trophoblasts were treated with different concentrations of progesterone for 48 h. Cell counts, steroidogenesis, and relevant gene and protein expression levels were measured. Progesterone inhibited trophoblast proliferation in a dose-dependent manner. High doses of progesterone significantly altered the expression levels of apoptosis-related and steroidogenesis-related genes and proteins, while low doses had a less pronounced effect. Thus, increased progesterone induces the apoptosis of porcine placental trophoblasts and induces abnormal steroidogenesis in the placenta. We believe that our study makes a significant contribution to the literature because it elucidates the effects of progesterone on porcine placental trophoblast functions.

**Abstract:**

Placentation and placental steroidogenesis are important for pregnancy and maternal–fetal health. As pregnancy progresses, the main site of progesterone (P4) synthesis changes from the corpus luteum to the placenta, in which placental trophoblasts are the main cell type for P4 synthesis. Therefore, this study investigated the effects of P4 on apoptosis and steroidogenesis in porcine placental trophoblasts and the underlying molecular mechanisms. Porcine placental trophoblasts were treated with different concentrations of P4 for 48 h in a serum-free medium in vitro. Cell number, steroidogenesis, and relevant gene and protein expression levels were detected. A high dose of P4 (10.0 μM) significantly increased P4 (*p* < 0.01), androstenedione (*p* < 0.05), testosterone (*p* < 0.05), and estradiol (*p* < 0.05) production in porcine placental trophoblasts compared with that in control cells, while a low dose of P4 (1 × 10^−3^ μΜ) had no marked impact on steroid production. The mRNA expression of apoptosis-related genes (*CASP3*, *CASP8*, and *Bax*) (*p* < 0.05) and steroidogenesis-related genes (*CYP11A1*, *CYP19A1*, and *StAR*) (*p* < 0.01) was upregulated, and the expression of *HSD3B* and *HSD17B4* was inhibited (*p* < 0.05) in the porcine placental trophoblasts treated with high doses of P4. Low doses of P4 had a lighter effect on gene expression than high doses. The expression of apoptosis-related proteins CASP3 (*p* < 0.05), and Bax (*p* < 0.01) and steroidogenesis-related proteins CYP19A1 (*p* < 0.05) and StAR (*p* < 0.01) was raised, but the proliferation-related protein CCND2 (*p* < 0.01) was downregulated in the pTr cells treated with high dose of P4. In comparison, a low dose of P4 inhibited the expression of Bax, CYP11A1 (all *p* < 0.01), and CCND2 (*p* < 0.05), but the expression of CASP3 (*p* < 0.05) and StAR (*p* < 0.01) was upregulated. In summary, excessive P4 can induce the apoptosis of porcine placental trophoblasts and lead to abnormal steroidogenesis in the placenta and hormone imbalance.

## 1. Introduction

Placental steroid hormones (progestogen, estrogen, testosterone, androgen, etc.) are mainly secreted by placental trophoblasts and are important biomarkers of pregnancy-associated diseases. They have a pivotal role in the maintenance of pregnancy, maternal adaptation to pregnancy, and fetal development [1,2]. Porcine placental trophoblast (pTr) cells are the chief cell types of the porcine placenta that perform endocrine functions and are an ideal model for studying the placental function and cell proliferation, migration, invasiveness, and steroid synthesis [3,4,5,6]. In early pregnancy, pTr cells adhere closely to maternal endometrial epithelial cells, forming the only bridge for maternal–fetal exchange [7,8]. Many nutrient transporters that affect placental nutrient transport efficiency are expressed in pTr cells and are regulated by maternal hormones, growth factors, and cytokines, suggesting that hormone levels may be related to placental nutrient transfer functions [9,10]. The structure of placental trophoblasts varies according to species and pregnancy period. Porcine placental trophoblasts consist of mononuclear trophoblast cells throughout pregnancy with no invasive capability, and only pigs and whales exhibit this feature [2,8]. Although the placental structures of interspecies are diverse, there are many common elements, especially those related to steroidogenesis and steroid hormone metabolism [11].

Progesterone is critical for the regulation of embryonic implantation [12] and promotes endometrial stromal differentiation, glandular secretion, and placentation during pregnancy [13]. With increasing gestational age, the placenta gradually replaces the ovary to become the largest organ for P4 secretion [14]. Except for humans, other mammals (mice, etc.) have higher progesterone levels in the early stages of fetal development. It is known that progesterone levels begin to decrease gradually in the middle and late stages of pregnancy [15,16,17]. However, there are reports that fetal blood P4 levels were independent of fetal age [18]. P4 can effectively maintain the growth and development of embryos, but excessive or insufficient P4 may lead to adverse pregnancy outcomes, such as threatened abortion [19]. P4 supplementation in early pregnancy is widely used to avoid abortion and prevent premature delivery, but changes in maternal P4 can also affect fetal P4 levels. For example, the application of exogenous P4 to the mother can significantly increase fetal serum P4 levels. Studies have found that P4 can promote uterine and placental angiogenesis, and when upregulated by trophoblast cells, it can express placental growth factor (PGIF), which is homologous to VEGF [15,20,21]. The secretory endometrium is vascular and glandular, and a lack of P4 can inhibit the transition of the endometrium to the secretory state [22]. P4 regulates the function of the exosomes derived from trophoblast cells to control intimal receptivity, ensuring the normal development of embryos during pregnancy [23]. P4 stimulates endometrial biosynthesis and promotes pregnancy via paracrine effects [24,25]. Endometrial stromal cells have been reported to increase the inhibitory effect of P4 on the proliferation of endometrial cancer cells through paracrine signal transduction; however, the mechanism is unclear [26]. The various biological effects of P4 are mainly mediated by the interaction between P4 and its receptor (PGR) [27], which promotes endometrial decidualization and maintains pregnancy mainly through its endocrine and immune functions [28]. Lissauer et al. [29] showed that P4 at a concentration of 10 μM had a more significant response to immune effects and inhibited the proliferation of human maternal T cells. The maternal immune system recognizes fetal antigens at the decidual–trophoblast interface [30], but the effect of P4 on pTr cell function remains unclear.

Therefore, this study intended to (1) assess the effects of the different concentrations of P4 on apoptosis and steroidogenesis in pTr cells and (2) assess the effect of P4 on relevant gene and protein expressions in pTr cells.

## 2. Materials and Methods

### 2.1. Ethics Statement

Living animals were not used in this study; therefore, ethical approval was not required.

### 2.2. Reagents

The following consumables were used in the cell culture: P4 and penicillin/streptomycin were obtained from Sigma-Aldrich (Burlington, MA, USA), Dulbecco’s modified Eagle’s medium (DMEM)/F-12, fetal bovine serum (FBS), and a 0.25% trypsin solution were obtained from Thermo Fisher Scientific (Waltham, MA, USA), and insulin–transferrin–selenium (ITS, 100×) was obtained from ScienCell (Carlsbad, CA, USA).

### 2.3. Cell Culture

Immortalized pTr cells (stem from Texas A&M University (College Station, TX, USA)) were grown in a DMEM/F-12 medium, with minor modifications, as previously reported [31]. The cells were cultured in dishes (d = 10 cm; Corning Inc., Corning, NY, USA) using 10 mL of DMEM/F-12 supplemented with FBS (10%), penicillin/streptomycin (1%), and ITS (1%) [8,32]. The medium was substituted every 48 h. When the confluence of cells reached approximately 80%, cells were collected in a 0.25% trypsin solution. After cell counting, an average of 1.0 × 10^6^ cells were implanted into cell culture plates (12-well; Corning) with 2 mL of the medium (containing FBS, penicillin/streptomycin, and ITS) and cultured in an environment of 5% CO_2_ and 95% air at 37 °C for the first 48 h, with a medium change at 24 h. Then, according to the specific experiment, they were washed twice with a 1 mL serum-free medium (containing penicillin/streptomycin and ITS) and were treated in that serum-free medium for 48 h.

### 2.4. Enzyme-Linked Immunosorbent Assay (ELISA) and Cell Counting

After treatment, ELISA kits (Jinenlai Biotech, Beijing, China) were used to detect the concentrations of four placental steroid hormones: P4 (GEL4686), androstenedione (A4, F8259), testosterone (T, GEL4562), and estradiol (E2; GEL4632). The sensitivities of the ELISA kit for P4, A4, T, and E2 were 80 pM, 30 pg/mL, 6 nM, and 8 pM, respectively. The average intra-assay coefficient of variation was 8.6–9.9%, and the average inter-assay coefficient was 9.4–11.1%.

For cell counting, the medium was gently removed from the wells, and the cells were rinsed with PBS, digested with trypsin, collected, and counted with an automatic cell counter (TZ20^TM^; Bio-Rad, Hercules, CA, USA) to count as previously described [33].

### 2.5. RNA Extraction and Quantitative Reverse-Transcriptase PCR (RT-qPCR)

The total RNA was extracted from cells using an RNAzol^®^ RT reagent (1 mL/sample; Molecular Research Center, Inc., Cincinnati, OH, USA). The RNA was dissolved in DEPC-treated water (Tiangen Biotech, Beijing, China), quantified using an Eppendorf BioSpectrometer Kinetic (Eppendorf, Hamburg, Germany) at 260 nm, diluted to 300 ng/μL, and stored at −80 °C.

The primers for the amplification of cell-proliferation-related genes (*CCND1*, *CCND2*, *Bax*, and *CDK4*), apoptosis-related genes (*CASP3*, *CASP8*, and *Bcl−2*), steroidogenesis-related genes (*CYP11A1*, *HSD17B4*, *HSD3B*, *StAR*, and *CYP19A1*), and *PGR* were designed or retrieved according to the gene sequence of pigs. Primer sequences and estimated amplified fragments are listed in Table 1.

The total RNA extracted was reverse-transcribed into cDNA in a 10 μL reaction system by using a Script cDNA Synthesis Kit (Bio-Rad), and then the first-strand cDNA was directly used for RT-qPCR or stored at −80 °C [34].

RT-qPCR was conducted using a CFX96 Touch Real-Time PCR detection system (Bio-Rad) containing iTaq^TM^ Universal SYBR^®^ Green SuperMix (Bio-Rad) in a 10 µL reaction system [35]. The PCR procedure was the same as previously described [32]. Additionally, two negative controls, namely a no-template control and a no-reverse-transcriptase control, were included to confirm the absence of contaminants in the master mix and DNA contamination in RNA, respectively [34]. The relative abundance of the target mRNA transcripts was calculated as 2^−^^∆∆Ct^ using the comparative threshold cycling method, normalized to *GAPDH* ribosomal RNA levels as previously described [34,35].

**Table 1:** Primers for target genes of pTr cells.

Gene	Sequence of Primers (5′ to 3′)	Fragment (bp)	GenBank ID	Reference
*CCND1*	F: GACCGCTTCCTGTCCCTGR: GTGGCACAGAGGGCGACGA	317	XM_021082686	[8]
*CCND2*	F: CGTCCAAGCTCAAAGAGACCR: CGAAGAATGTGCTCGATGAA	169	NM_214088	[36]
*CDK4*	F: GCATCCCAATGTTGTCCGR: GGGGTGCCTTGTCCAGATA	125	NM_001123097	[8]
*CASP8*	F: TCCTGAGCCTGGACTACATR: CTCCTCCTCATTGGTTTCC	185	NM_001031779.2	[8]
*CASP3*	F: GCCATGGTGAAGAAGGAAAAR: GTCCGTCTCAATCCCACAGT	167	NM_214131	[8]
*StAR*	F: GGAAAAGACACAGTCATCACCCATR: CAGCCAGCACACACACGGAAC	121	NM_213755.2	[8]
*HSD17B4*	F: TGCCATGAGAGTTGTGAGGAAAR: CCTCAGGAGTCATTGGCTGATT	127	XM_021081514.1	[8]
*HSD3B*	F: TCCACACCAGCAGCATAGAGR: ATACATGGGCCTCAGAGCAC	206	NM_001004049.2	[8]
*CYP19A1*	F: GTATATCGCCATGGTCATGR: AGCAGGCCGCTGGTCTCAT	144	NM_214429.1	[8]
*CYP11A1*	F: GCCGCATGGGACACTATTTTR: ATTTCCCAGGAGGCGGTAGA	120	NM_214427.1	[8]
*Bax*	F: AAGCGCATTGGAGATGAACTR: CGATCTCGAAGGAAGTCCAG	251	XM_003127290.5	[37]
*Bcl*–*2*	F: TGTGTGGAGAGCGTCAACCGR: CCCATACAGCTCCACAAAGGCAT	138	XM_021099593.1	[38]
*PGR*	F: GATTCAGAAGCCAGCCAGAGR: GATGCTTCATCCCCACAGAT	83	GQ903679	[39]
*GAPDH*	F: AAGGAGTAAGAGCCCCTGGAR: TCTGGGATGGAAACTGGAA	140	NM_001206359.1	[8]

### 2.6. Western Blot Analysis

After treatment, the proteins were obtained according to cell lysate instructions (RIPA, R1091, Lablead, Beijing, China), and protein concentrations were determined using a BCA protein assay kit (B5000, Lablead, Beijing, China). Equal amounts of protein extracts (25 μg) were separated on SDS–PAGE gels and then transferred to polyvinylidene difluoride (PVDF) membranes. The membranes were sealed in a blocking solution (P0252, Beyotime, Shanghai, China) for 30 min at room temperature and then incubated with the indicted primary antibodies at 4 °C, and the antibodies used in Western blot experiments are listed in Table 2. Then, the membranes were hatched with secondary antibodies (diluted with the blocking solution for 1:5000) for 1 h at room temperature. GAPDH or β-actin was used as the internal reference. The protein bands were developed using a gel imaging system (Bio-Rad) and quantitative analysis using ImageJ (NIH, USA).

### 2.7. Experimental Design

Experiment 1 was designed to evaluate the effects of the different concentrations of P4 on the cell proliferation of pTr cells and the production of steroid hormones (P4, A4, T, and E2). The cells were cultured in a 10% fetal bovine serum medium for 48 h and washed twice with a 1 mL serum-free medium, and one of the following treatments was applied: 0.1 × 10^−6^ μM, 1 × 10^−5^ μM, 1 × 10^−4^ μM, 1 × 10^−3^ μM, 1 × 10^−2^ μM, 0.1 μM, or 10 μM of P4 for 48 h [8,32,40]. The cells were counted when the treatments were finished. The supernatants were collected to detect steroidogenesis. Based on their cell number, pTr cells were treated with a high dose (10 μM) or low dose (1 × 10^−3^ μM) of P4 for the effect of P4 on gene and protein expression in the pTr cells.

In Experiment 2, we intended to study the effects of P4 on pTr-cell-proliferation-related (*CCND1*, *CCND2*, *Bax*, and *CDK4*), apoptosis-related (*CASP3*, *CASP8*, and *Bcl−2*), steroid-synthesis-related (*CYP11A1*, *CYP19A1*, *HSD17B4*, *HSD3B*, and *StAR*), and *PGR* genes and relevant proteins. The cell culture was as described in Experiment 1; the cells were treated with either 1 × 10^−3^ μM or 10 μM P4 for 48 h, lysed, and subjected to RNA and protein extraction. The control group was treated with the same equivalence of the serum-free medium as the experimental group.

### 2.8. Statistical Analysis

After treatment, P4, A4, T, and E2 production in the cell medium were expressed as pM/mL, pg/mL, nM/mL, and pM/mL per 10^5^ cells, respectively; these values were calculated as the number of cells at the end of treatment. Each experiment used three different pTr wells as experimental replicates, and each treatment was repeated three times. If the data did not distribute to a normal population, the log-transformation data were used for statistical analyses. The treatment effects on dependent variables (cell number, hormone production, target gene relative mRNA transcript abundance, and protein expression) were statistically analyzed using SPSS 25.0. The treatment effects were analyzed using ANOVA, and the data are shown as the mean ± SEM. Statistical significance was set at *p* < 0.05 and *p* < 0.01. All bar charts were produced using GraphPad Prism 8.0.2.

## 3. Results

### 3.1. Effect of P4 on Proliferation of pTr Cells

**Figure 1:**
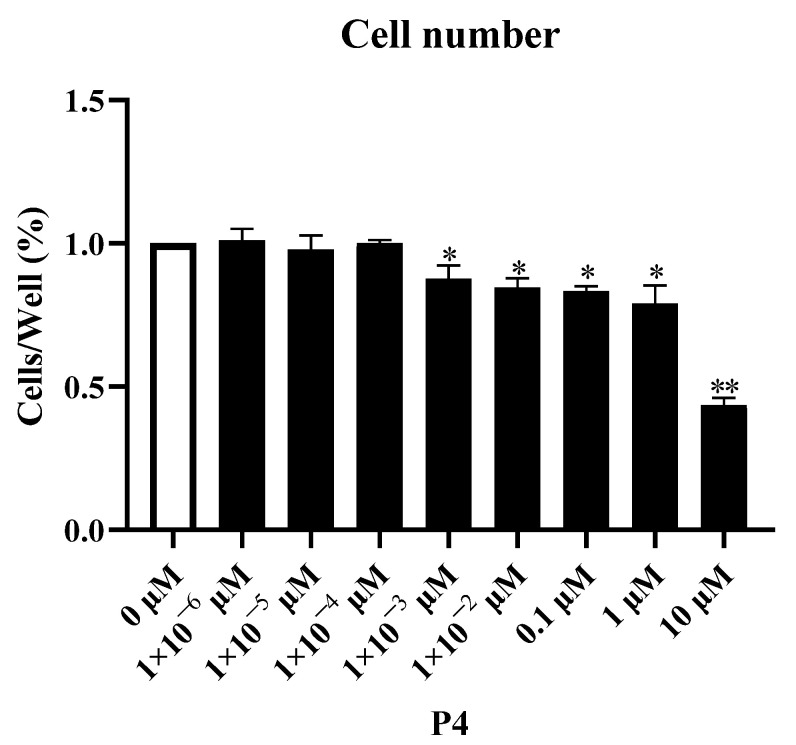
Dose–response effect of P4 on porcine placental trophoblast cell numbers. The results represent the average ± SEM of three independent experiments; * indicates values significantly different from that of the control group (*p* < 0.05); ** indicates values significantly different from that of the control group (*p* < 0.01).

P4 inhibited pTr survival in a dose−dependent manner (Figure 1). After 48 h of treatment, the survival rate decreased as the P4 treatment concentration increased. The number of pTr cells decreased significantly when the P4 concentration exceeded 1 × 10^−3^ μM (*p* < 0.05). The cell counts decreased by 12.3%, 15.4%, 16.7%, and 21.0% at 1 × 10^−3^ μM, 0.01 μM, 0.1 μM, and 1 μM (*p* < 0.05), respectively, and decreased by 56.5% at 10.0 μM (*p* < 0.01), respectively. According to the observed changes in the cell, a P4 dose with a mild inhibitory effect (1 × 10^−3^ μM) was selected as the low dose, and 10.0 μM of P4 was selected as the high dose in the following experiments.

### 3.2. Effect of P4 on Steroid Hormone Synthesis in pTr Cells

**Figure 2:**
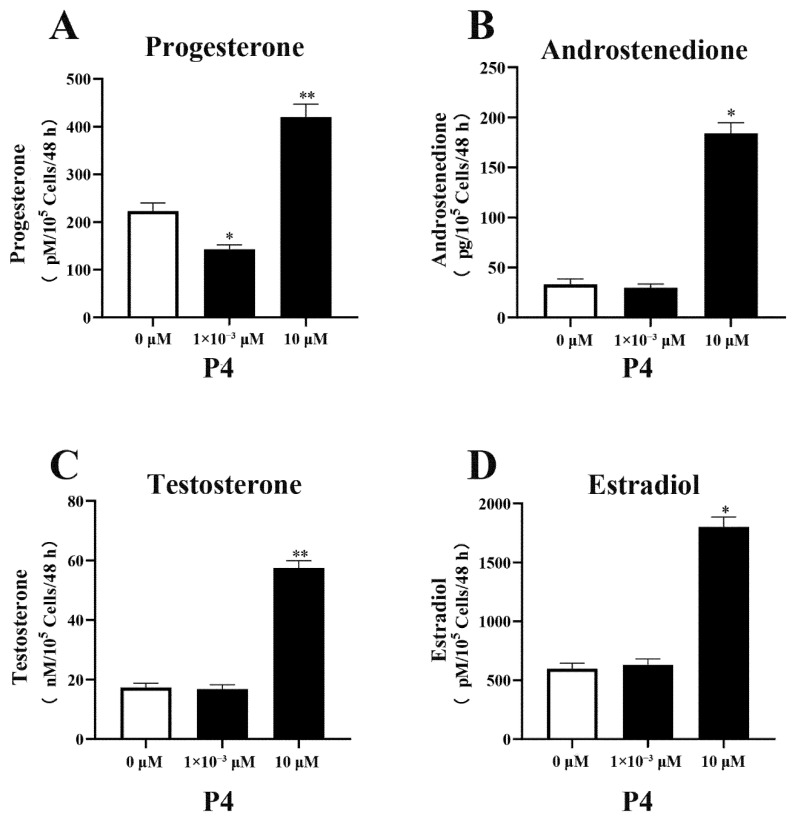
Effect of P4 on P4 (**A**), A4 (**B**), T (**C**), and E2 (**D**) levels in pTr cells after 48 h treatment. The results represent the average ± SEM of three independent experiments; * indicates values significantly different from that of the control group (*p* < 0.05); ** indicates values significantly different from that of the control group (*p* < 0.01).

P4 had a significant indigenous effect on steroidogenesis in pTr cells, especially at high doses (Figure 2). Compared with the control group (without P4) and treatment with a low dose of P4, the high dose (10.0 μM) of P4 promoted steroidogenesis in the pTr cells. The production of P4, A4, T, and E2 increased by 89% (*p* < 0.01), 434% (*p* < 0.05), 231% (*p* < 0.01), and 201% (*p* < 0.05), respectively. Low doses of P4 inhibited P4 production in pTr cells (*p* < 0.05) but had no significant effect on the synthesis of other hormones (*p* > 0.05).

### 3.3. Effects of P4 on Gene Expression in pTr Cells

#### 3.3.1. Cell-Proliferation-/Apoptosis-Related Gene Expression

**Figure 3:**
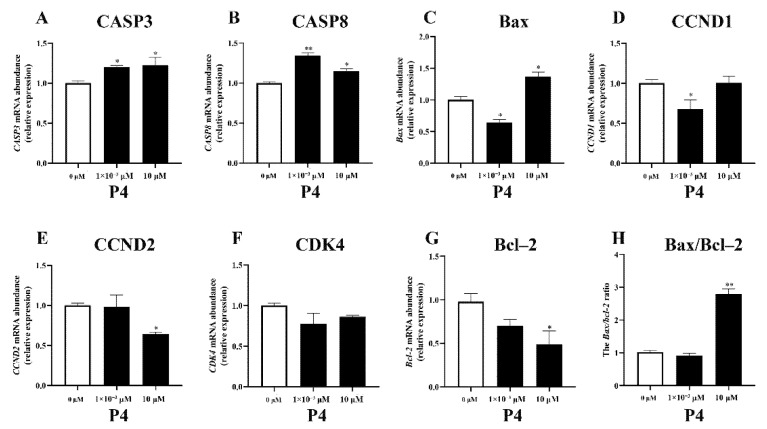
Effects of P4 on mRNA transcription abundance of apoptosis-related genes (**A**–**C**), proliferation-related genes (**D**–**G**), and *Bax/Bcl*–*2* ratio (**H**) in pTr cells treated with P4 for 48 h. The results represent the average ± SEM of three independent experiments; * indicates values significantly different from that of the control group (*p* < 0.05); ** indicates values significantly different from that of the control group (*p* < 0.01).

The different concentrations of P4 had different effects on the expression of genes related to proliferation and apoptosis in pTr cells (Figure 3). The administration of P4 increased the expression of the apoptotic genes *CASP3* and *CASP8* (*p* < 0.05); only a high dose of P4 increased *Bax* expression, while a low dose of P4 inhibited *Bax* expression (*p* < 0.05). Regarding cell-proliferation-related genes, low doses of P4 inhibited *CCND1* expression (*p* < 0.05), and high-dose P4 significantly inhibited *CCND2* and *Bcl−2* gene expression (*p* < 0.05) but had no significant effect on *CCND1* expression (*p* > 0.05). P4 treatment had no significant effect on *CDK4* expression (*p* > 0.05). High-dose P4 significantly increased the *Bax/Bcl−2* ratio in pTr cells (*p* < 0.01).

#### 3.3.2. Expression of Steroidogenesis-Related Genes

**Figure 4:**
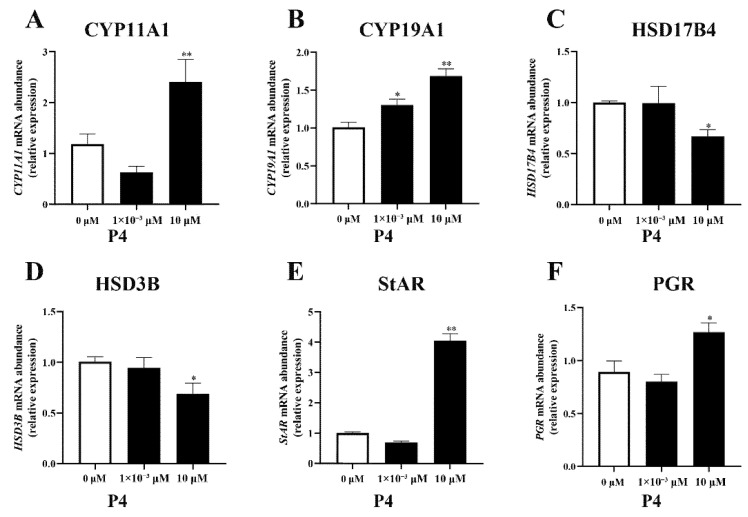
Effect of P4 on mRNA abundance of steroidogenesis−related genes *CYP11A1* (**A**), *CYP19A1* (**B**), *HSD17B4* (**C**), *HSD3B* (**D**), *StAR* (**E**), and *PGR* (**F**) mRNA transcripts in pTr cells after 48 h treatment. The results represent the average ± SEM of three independent experiments; * indicates values significantly different from that of the control group (*p* < 0.05); ** indicates values significantly different from that of the control group (*p* < 0.01).

The different concentrations of P4 had different effects on the expression of steroidogenesis-related genes in pTr cells (Figure 4). A high dose of P4 increased the expression of *CYP11A1*, *CYP19A1*, *StAR* (all *p* < 0.01), and *PGR* (*p* < 0.05) and inhibited the expression of *HSD3B* and *HSD17B4* (*p* < 0.05). A low dose of P4 had no significant effect on gene expression, except on the *CYP19A1* gene, suggesting that *CYP19A1* gene expression may be sensitive to P4.

### 3.4. Effects of P4 on Protein Expression in pTr Cells

**Figure 5:**
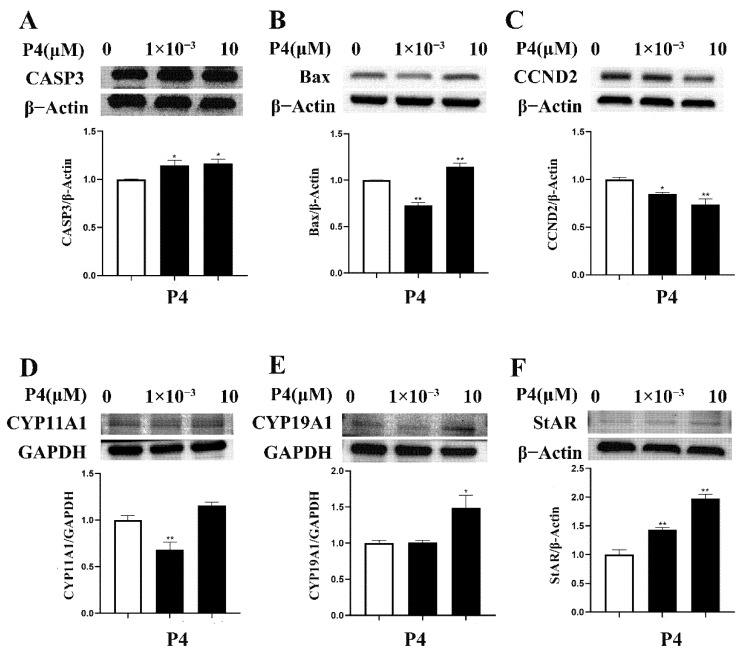
Effect of P4 on protein abundance of CASP3 (**A**), Bax (**B**), CCND2 (**C**), CYP11A1 (**D**), CYP19A1 (**E**), and StAR (**F**) in pTr cells after 48 h treatment. The results represent the average ± SEM of three independent experiments; * indicates values significantly different from that of the control group (*p* < 0.05); ** indicates values significantly different from that of the control group (*p* < 0.01). Original Western blot bands and intensity ratio are shown in Figure S1 and Table S1.

The different concentrations of P4 had different effects on protein expression in pTr cells (Figure 5). A high dose of P4 enhanced the protein abundance of CASP3, CYP19A1 (all *p* < 0.05), and Bax, StAR (all *p* < 0.01) and inhibited CCND2 (*p* < 0.01). A low dose of P4 enhanced the protein of CASP3 (*p* < 0.05) and StAR (*p* < 0.01) but inhibited Bax, CYP11A1 (all *p* < 0.01), and CCND2 (*p* < 0.05).

## 4. Discussion

In this research, pTr cells were challenged with the different concentrations of P4 to preliminarily investigate the effect of P4 on cell proliferation as well as steroidogenesis. P4 concentration was selected based on the results of this study and from the published literature [41,42,43]. The results showed that a high dose of P4 induced apoptosis in pTr cells and upregulated the expression of steroid-hormone-synthesis-related genes, resulting in increased steroidogenesis in pTr cells in vitro.

CYP11A1 is the initial step in catalyzing steroid biosynthesis, and StAR facilitates cholesterol delivery from the adventitia to the intima [44]. StAR transports cholesterol to the mitochondria, where CYP11A1 converts it to pregnenolone, which synthesizes steroid hormones in response to the enzymes involved in steroid synthesis [45]. In this study, in order to explain how P4 regulates steroid synthesis in pTr cells, RT-qPCR was used to quantify the mRNA transcription abundance of *CYP11A1*, *CYP19A1*, *HSD3B*, *HSD17B4*, and *StAR* genes, and Western blot was used to quantify the protein expression of CYP11A1, CYP19A1, and StAR. The results indicated that a high dose of P4 may affect the synthesis of steroid hormones in pTr cells by changing the expression of the genes and proteins related to steroid synthesis. Our study found that after a high-dose P4 treatment, P4 levels in pTr cells were significantly upregulated, and the corresponding expression of *StAR* and *CYP11A1* were also significant raised. Miao et al. [46] found that *StAR* expression was positively correlated with P4 levels in goat ovarian granulosa cells, which is consistent with our results in pTr cells. CYP11A1 converts cholesterol to P4 [47], suggesting that *StAR* and *CYP11A1* may jointly regulate P4 production. As the short half-life of progesterone [48], the added progesterone could be ignored in the final detection results. Increased A4 and T production are related to the increased expression of *StAR*, *CYP11A1*, and *HSD3B* [49,50]; however, our study found that *HSD3B* expression significantly decreased after the high-dose P4 treatment, which is inconsistent with previous studies and requires further study. Pregnenolone is a precursor of several steroid hormones (P4, A4, T, etc.), which are produced through CYP11A1 transformation in the inner membrane of the mitochondria [45,51,52,53,54]. However, the CYP11A1 was not augmented with a high dose of P4, indicating that a high dose of P4 may cause increases in P4, T, and A4 synthesis by affecting the gene and protein expression of StAR. HSD17B4 is not only related to steroid synthesis but also proved to be a novel proliferation-promoting gene, whose overexpression or knockout can promote or inhibit, respectively, the proliferation of the human hepatocellular carcinoma cell line HepG2 [55]. Therefore, the inhibition of pTr proliferation by high doses of P4 may also be related to a decrease in *HSD17B4* expression. CYP19A1 is a key enzyme in estrogen synthesis and is related to the mRNA expression of proliferation-related genes (*CCND1* and *CDK2*). When *CYP19A1* was knocked down, proliferation-related genes were upregulated [56], which provides another explanation for the inhibition of proliferation-related genes in pTr cells after P4 treatment. In addition, the placenta is the main site of estrogen production and the most active site for *CYP19A1* expression [57]. Decreased estrogen levels are associated with downregulated *CYP19A1* expression in mouse granulosa cells [58]. Our results showed that a high dose of P4 promoted E2 synthesis, possibly due to the upregulation of *CYP19A1* gene expression. This is consistent with our research indicating that a high dose of P4 promoted E2 synthesis in pTr cells and upregulated the gene and protein expression of CYP19A1, suggesting that E2 synthesis is mainly related to the gene and protein expression of CYP19A1. During pregnancy, an appropriate increase in E2 contributes to placental vascular function, while excessive E2 secretion may lead to oxidative stress [59]. The imbalance of coordination between P4 and E2 may lead to inflammation and reduce the endometrial receptivity to the embryo. In early pregnancy, E2 induces endometrial epithelial proliferation, while P4 inhibits E2-induced proliferation [60]. Studies have reported that the decrease in P4 before delivery may be due to the transformation of P4 into E2 in the placenta, which may be related to species [28].

P4 inhibits proliferation and induces the apoptosis of breast cancer cells at relatively high physiological concentrations (approximately 10 μM) [40]. Studies have shown that P4 can inhibit the invasion of human trophoblast cells and reduce the number of invasive cells with the increase in P4 [61]. In addition, P4 inhibits the proliferation of endometrial cancer cells via a paracrine action [26]. Horita et al. [62] reported that P4 increased *p53* gene expression and induced apoptosis in breast cancer cells. The apoptotic cascade may be triggered by both the extrinsic and intrinsic pathways. In the extrinsic pathway, apoptotic factors (tumor necrosis factor, etc.) bind to membrane receptors and activate *CASP8* and *CASP3* to induce DNA fragmentation and cell death. In the intrinsic pathway, *CASP3* is activated by increasing the ratio of pro-apoptotic proteins (*Bax*) to anti-apoptotic proteins (*Bcl−2*) and finally induces apoptosis [63,64]. The results of the present study are consistent with these findings, indicating that a high dose of P4 may increase the *Bax*/*Bcl−2* ratio by changing the balance between *Bax* and *Bcl−2* and stimulate the upregulation of *CASP3* and *CASP8*, resulting in the apoptosis of pTr cells. Formby and Wiley [43] found that P4 inhibited the proliferation of breast cancer cells in a dose-dependent manner by activating the apoptotic pathway (*P53* was upregulated, and *Bcl−2* was downregulated) in vitro. Cyclin D1 (*CCND1*) is a G1/S phase-specific cell cycle regulator that promotes cell proliferation by binding and activating *CDK4* and *CDK6* [8]. In early pregnancy, *CCND1* and *CDK4* regulate cell cycle progression and promote the cell cycle transition from G1 to S in pTr cells [65]. It has been found that in the process of placentation, if adverse pregnancy occurs, the expression of *CCND1* is upregulated, such as in preeclampsia [66]. In this study, *CASP3* and *CASP8* gene expression significantly increased, *CCND1* and *CCND2* gene expression significantly decreased, and *CDK4* did not show significant changes in the pTr cells treated with high doses of P4. It is speculated that a high dose of P4 inhibits cell cycle progression by reducing the expression of *CCND1*, suppressing its interaction with *CDK4*, and leading to cell death by increasing the expression of *CASP3* and *CASP8*. In addition, P4 upregulated the expression levels of apoptosis-related proteins (CASP3 and Bax), inhibited the expression of proliferation-related protein (CCND2), suggesting that P4 induced the apoptosis of pTr cells probably because it upregulated the expression of apoptosis-related genes and proteins and downregulated the expression of proliferation-related genes and proteins.

There is a complex endocrine–paracrine–autocrine regulatory system in the placenta [67,68]. As pregnancy progresses, the placenta becomes the principal source of steroid hormone synthesis [8,32]. An imbalance of steroid hormones is related to many pregnancy complications, such as preeclampsia, caused by insufficient P4 [69]. A premature increase in P4 levels is related to abnormal implantation and decreased pregnancy rates [70,71,72,73]. Due to the paracrine effect of P4 on endometrial cells, the proliferation of endometrial cancer cells is inhibited [26,74]. P4 generally performs genomic and non-genomic functions by inducing *PGR* gene expression and activating its receptors [75]. This study detected that high doses of P4 upregulated the gene expression of *PGR*, suggesting that P4 binds to its receptor to regulate pTr cell function.

## 5. Conclusions

According to the results of the current study, a high dose of P4 can increase steroidogenesis through the upregulation of the expression of steroid-hormone-synthesis-related genes and proteins, thus inducing apoptosis in pTr cells.

## Figures and Tables

**Table 2:** Primary antibodies used in Western blot experiments.

Antibody	Vendor	Code	Host Species	Dilution
CASP3	Abclonal	A2156	Rabbit	1:1000
Bax	Cell Signaling Technology	2772T	Rabbit	1:1000
CCND2	Proteintech	10934-1-AP	Rabbit	1:1000
CYP11A1	Absin	abs120402	Rabbit	1:1000
CYP19A1	Absin	122200	Rabbit	1:1000
StAR	Proteintech	12225-1-AP	Rabbit	1:1000
GAPDH	Abclonal	AC002	Mouse	1:5000
β–Actin	Proteintech	66009-1	Mouse	1:5000

## Data Availability

Data sharing is not applicable to this article.

## Supplementary Materials

The following supporting information can be downloaded at: https://www.mdpi.com/article/10.3390/ani12192704/s1, Figure S1: Effect of P4 on protein abundance of CASP3 (A), Bax (B), CCND2 (C), CYP11A1 (D), CYP19A1 (E), and StAR (F) in pTr cells after 48 h treatment. Table S1: The intensity ratio of bands in Western blot experiments.
