# Supplementary material for: Progesterone Induces Apoptosis and Steroidogenesis in Porcine Placental Trophoblasts
Source: Animals (Basel). 2022 Oct 8;12(19):2704. doi: 10.3390/ani12192704 (PMC9558511; doi:10.3390/ani12192704)
Supplement: Supplementary file 1 [file animals-12-02704-s001.zip › Supplementary Materials Figure S1.pdf]

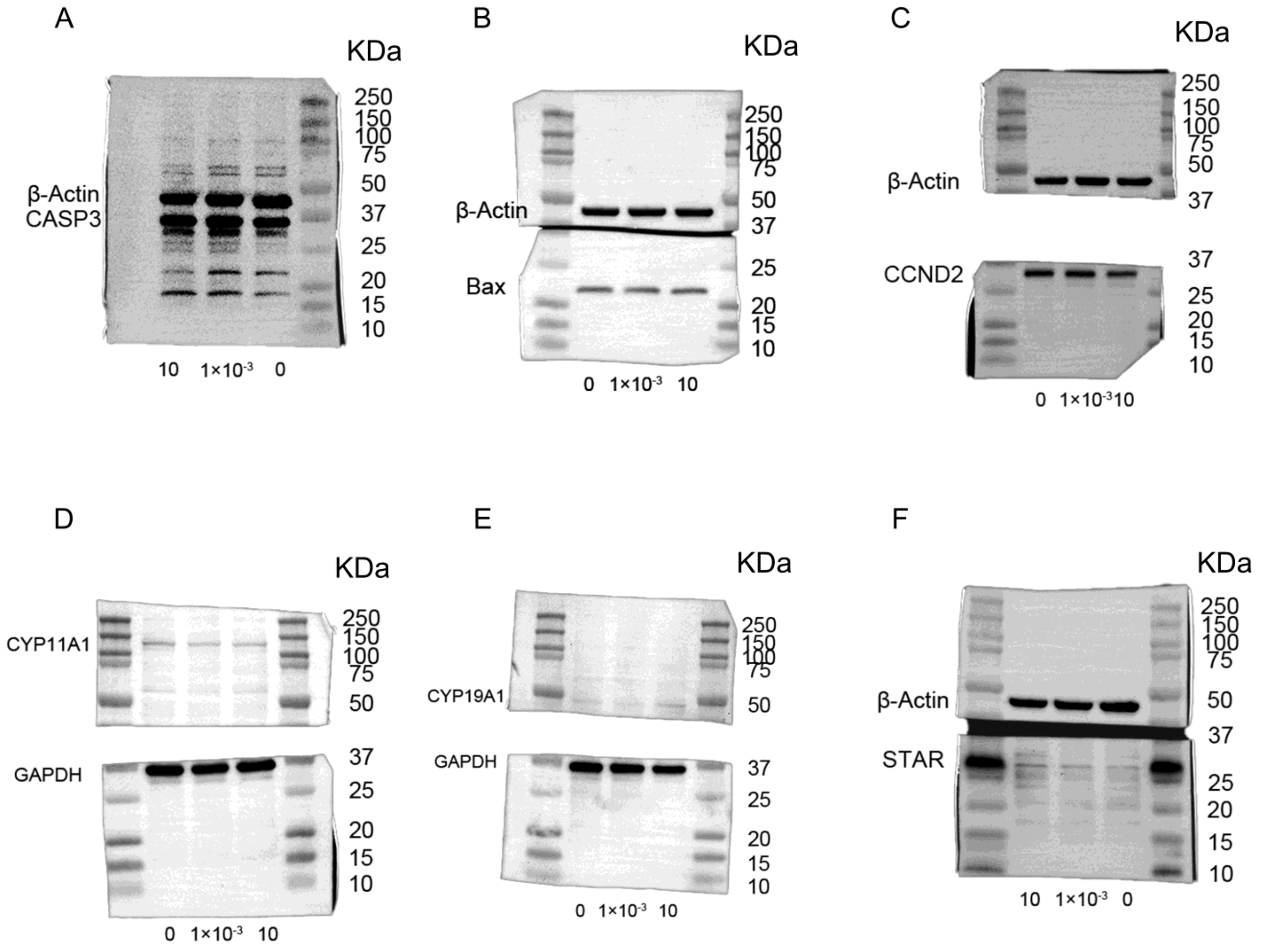

Figure S1. Effect of P4 on protein abundance of CASP3 (A), Bax (B), CCND2 (C), CYP11A1 (D), CYP19A1 (E), and StAR (F) in pTr cells after 48 h treatment.
