# Supplementary material for: Progesterone Induces Apoptosis and Steroidogenesis in Porcine Placental Trophoblasts
Source: Animals (Basel). 2022 Oct 8;12(19):2704. doi: 10.3390/ani12192704 (PMC9558511; doi:10.3390/ani12192704)
Supplement: Supplementary file 1 [file animals-12-02704-s001.zip › Supplementary Materials Table S1.pdf]

Table S1. The intensity ratio of bands in Western blot experiments.

Treatment	Band intensity		Intensity ratio	Protein abundance
	**CASP3**	**β–Actin**	**CASP3/β–Actin**	
Control	341270	443888	0.77	1.00
P4 (1×10-3 μM)	404782	422473	0.96	1.25
P4 (10 μM)	399211	394300	1.01	1.32
	**BAX**	**β–Actin**	**BAX/β–Actin**	
Control	124535	328084	0.38	1.00
P4 (1×10-3 μM)	95207	357027	0.27	0.70
P4 (10 μM)	143740	342786	0.42	1.10
	**CCND2**	**β–Actin**	**CCND2/β–Actin**	
Control	222318	312558	0.71	1.00
P4 (1×10-3 μM)	218436	354039	0.62	0.87
P4 (10 μM)	150739	336999	0.45	0.63
	CYP11A1	**GAPDH**	**CYP11A1/GAPDH**	
Control	22345	351696	0.06	1.00
P4 (1×10-3 μM)	15654	325300	0.05	0.76
P4 (10 μM)	25199	348850	0.07	1.14
	CYP19A1	**GAPDH**	**CYP19A1/GAPDH**	
Control	24644	380156	0.06	1.00
P4 (1×10-3 μM)	21838	358194	0.06	0.94
P4 (10 μM)	33146	311988	0.11	1.64
	StAR	**β–Actin**	StAR/**β–Actin**	
Control	23793	344203	0.07	1.00
P4 (1×10-3 μM)	27193	287971	0.09	1.37
P4 (10 μM)	41507	285052	0.15	2.11
